# Anomalous HIV-1 RNA, How Cap-Methylation Segregates Viral Transcripts by Form and Function

**DOI:** 10.3390/v14050935

**Published:** 2022-04-29

**Authors:** Kathleen Boris-Lawrie, Gatikrushna Singh, Patrick S. Osmer, Dora Zucko, Seth Staller, Xiao Heng

**Affiliations:** 1 Department of Veterinary and Biomedical Sciences, University of Minnesota, Saint Paul, MN 55108, USA; gsingh@umn.edu (G.S.); zucko001@umn.edu (D.Z.); 2 Department of Neurosurgery, University of Minnesota, Minneapolis, MN 55455, USA; 3 Department of Astronomy, The Ohio State University, Columbus, OH 43210, USA; osmer.1@osu.edu; 4 Department of Biochemistry, University of Missouri, Columbia, MO 65211, USA; saspn4@mail.missouri.edu

**Keywords:** epigenetic modification, internal ribosome entry, junD, NCBP3, RNA virus, ribosome scanning, specialized translation, trimethylguanosine (TMG) cap

## Abstract

The acquisition of m^7^G-cap-binding proteins is now recognized as a major variable driving the form and function of host RNAs. This manuscript compares the 5′-cap-RNA binding proteins that engage HIV-1 precursor RNAs, host mRNAs, small nuclear (sn)- and small nucleolar (sno) RNAs and sort into disparate RNA-fate pathways. Before completion of the transcription cycle, the transcription start site of nascent class II RNAs is appended to a non-templated guanosine that is methylated (m^7^G-cap) and bound by hetero-dimeric CBP80-CBP20 cap binding complex (CBC). The CBC is a nexus for the co-transcriptional processing of precursor RNAs to mRNAs and the snRNA and snoRNA of spliceosomal and ribosomal ribonucleoproteins (RNPs). Just as sn/sno-RNAs experience hyper-methylation of m^7^G-cap to trimethylguanosine (TMG)-cap, so do select HIV RNAs and an emerging cohort of mRNAs. TMG-cap is blocked from Watson:Crick base pairing and disqualified from participating in secondary structure. The HIV TMG-cap has been shown to license select viral transcripts for specialized cap-dependent translation initiation without eIF4E that is dependent upon CBP80/NCBP3. The exceptional activity of HIV precursor RNAs secures their access to maturation pathways of sn/snoRNAs, canonical and non-canonical host mRNAs in proper stoichiometry to execute the retroviral replication cycle.

## 1. Introduction

### 1.1. Anomalous HIV RNAs Are Indispensable to Carry out the Retroviral Replication Cycle

Hosts support every step of the HIV-1 (HIV) replication cycle [1]. Vast evidence has shown retroviral precursor RNAs subvert the mRNA metabolism pathways of hosts [2]. The subversive behavior of HIV RNAs originates from primary sequence content and dynamic intramolecular structural content [3]. By sorting of nascent HIV transcripts into host ribonucleoprotein (RNPs), HIV precursors engage host maturation pathways of both mRNAs and non-coding RNAs. As a result, the 9000 nucleotide (nt) HIV precursor RNA manifests exceptional coding capacity. The RNA-protein interactions necessary for the anomalous activity of HIV precursor RNA are the focus of this article.

### 1.2. The Biogenesis of HIV RNA Is Aligned with the Biogenesis of Host Noncoding RNA and Protein Coding RNA

The combined activity of RNA polymerase (RNAP) I, II and III is required to synthesize the host transcriptome. The majority of RNA products are noncoding RNAs and ultimately just 4% of the products become mRNA templates translated to protein [3]. Vastly outnumbered, the expression of the protein-coding transcripts requires RNA fate pathways that govern the maturation of noncoding RNAs [3].

RNAPII synthesizes precursor mRNAs, but most of its products are precursor small nuclear RNAs (snRNAs) and nucleolar RNAs (snoRNAs). Emerging from the same process of class II gene transcription, the noncoding RNAs enter distinct maturation pathways from protein-coding RNAs. They obtain unique epigenetic modifications and segregate with nucleolar RNPs that guide the assembly of massive RNA-protein machineries (spliceosomes and ribosomes) or cytoplasmic RNPs that translate mature mRNA. The hallmark events in the lives of snRNA, snoRNA and mRNA commence with co-transcriptional loading of similar RNA binding proteins, but come to fruition by different trafficking pathways and epigenetic modifications that license different RNA fates.

The HIV precursors share trafficking pathways with host sn/snoRNAs and mRNAs. One 9000 nucleotide full length HIV RNA encodes regulatory, accessory and structural proteins and dimeric genomic RNA. Full understanding of the anomalous RNA fates is a pressing goal in virology. Fulfillment is to have broad ramifications by exposing host aberrations affecting human health and originating new targets and RNA-directed therapeutics to cure HIV disease.

The functional plasticity of HIV-1 primary RNA has been attributed to shape-shifting of the 5′-untranslated region (UTR) [4,5]. Intense examinations have documented significant interplay between co-transcriptional host RNP assembly and the fate of mature HIV RNAs in productive infection [2]. Structural variables at the 5′-UTR of noncoding RNAs have been shown to affect function too [6,7,8]. For instance, snRNAs require nt-nt pairings within the 5′-Sm site [consensus PuA(U)_4–6_GPu], which engenders the hypermethylation of the 5′-cap (more below). The essential cis-acting structural motifs near the TSS of snoRNAs require nt-nt pairings within Box C (C′ in U3 snoRNA), which engender the hypermethylation of the snoRNA 5′-cap. The different energetically-tenable shapes within the 5′-UTR of sn/snoRNAs are necessary for the epigenetic modification of the 5′-cap.

## 2. HIV Precursor RNAs Enter Mutually Exclusive RNA-Fate Pathways during Transcription

### 2.1. Two-Phase Transcriptional Programming Originates at the HIV-1 Trans-Activation Responsive Element (TAR)

Early after HIV infection, proviral transcripts are alternatively spliced and completely processed into mRNAs that are translated to Tat and Rev regulatory proteins and Nef [9]. Tat RNA binding protein has particular specificity for the nascent TAR RNA structure (nts + 1 to 57) [10]. Tat/TAR interaction stimulates the epigenetic modification of the TSS, the release of RNAPII from pausing and the rate of new initiation at the HIV promoter [11]. Rev is an RNA binding protein with specific affinity for the Rev-responsive element (RRE) and also, the 5′-UTR (stem loop (SL) 1) [12]. Among its activities, Rev/RRE complex mediates the assembly of RNPs for nuclear export by the CRM1 nuclear export receptor and retains unspliced and singly spliced (US, SS) HIV transcripts in appropriate stoichiometry [11,13]. Despite CRM1 nuclear export activity is normally reserved for noncoding snRNAs and host proteins, the Rev/RRE-dependent US and SS mRNAs are licensed for translation. Just as important for HIV proliferation is that a small portion of US transcripts become dimerized and packaged into virions. Steps to reaching consensus on this virus-host interface are a goal molecular virologists [14].

### 2.2. HIV RNA Structures Formed by Heterogeneous 5′UTR nt-nt Pairings

**Figure 1:**
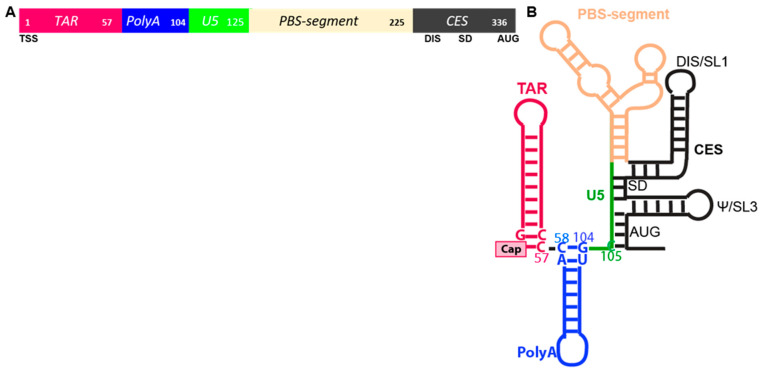
Motifs within the HIV 5′-untranslated region (UTR). (**A**) Linear arrangement of TAR, Tat trans-activation responsive element, magenta; PolyA, blue; U5, Unique 5′ region, green; PBS-segment containing the primer binding site (PBS), tan; CES, core encapsidation signal, black, Numbers indicate position in the 5′-UTR. Lower line: TSS, transcription start site; DIS, dimerization initiation site inclusive of SL1; SD, splice donor; AUG, gag start codon. (**B**) Secondary structure model. Select nucleotides pairings coordinate TAR-PolyA stem loops and PolyA-U5 stem.

Composed of heterogeneous structural motifs, the HIV 5′-UTR coordinates transcription, splicing, translation and packaging and also engenders the hallmark early replication events: reverse transcription and integration [4,5]. Within the 5′-terminal ~350 nucleotides (nt) of the precursor RNA, the RNA elements are the TAR stem-loop, metastable PolyA hairpin, unique (U5) region and segment containing tRNA primer binding site (PBS), splicing donor (SD), dimerization initiation site (DIS), core encapsidation signal (CES) and AUG start codon (Figure 1A). Mutations in the 5′-UTR have a direct impact on translation and packaging, but alter the overall folding of the RNA, thus complicating the dissection of either process [13,15].

RNA folding experiments with segments of the 5′-UTR have demonstrated biophysical equilibrium between nt–nt pairings fosters two predominant conformers that are monomeric and dimer-competent. By the analysis of nt–nt pairings that promote the intramolecular stability of either monomer or dimer-competent conformations in biochemical, biophysical and genetic experiments, consensus has been reached of structural motifs affecting viral replication events [16]. In the dimer-competent conformation, long range pairing of U5 with residues around the translation start codon exposes DIS for intermolecular dimerization (Figure 1B). Residues spanning the 5′-SD sequester in a three-way junction likely to preclude U1snRNP recognition and forestall alternative splicing. In the monomer conformation, U5 pairs with DIS, precluding intermolecular dimerization. Tertiary modeling from these experimental input constraints predicted metastable nt–nt pairings at the bottom of PolyA hairpin. For instance, U103C favored the monomer conformation [17]. In cells, U103C reduced the competitive packaging activity of the mutant RNA [17] and increased the translation rate [18], indicating that thermodynamic equilibrium can significantly influence the utility of the anomalous HIV RNA. Additional in-solution studies validated the U103C function change was attributable to altered host binding protein [19,20].

### 2.3. Co-Transcriptional Pause Is Coupled with the Covalent Modification of TSS and CBC Engagement

**Figure 2:**
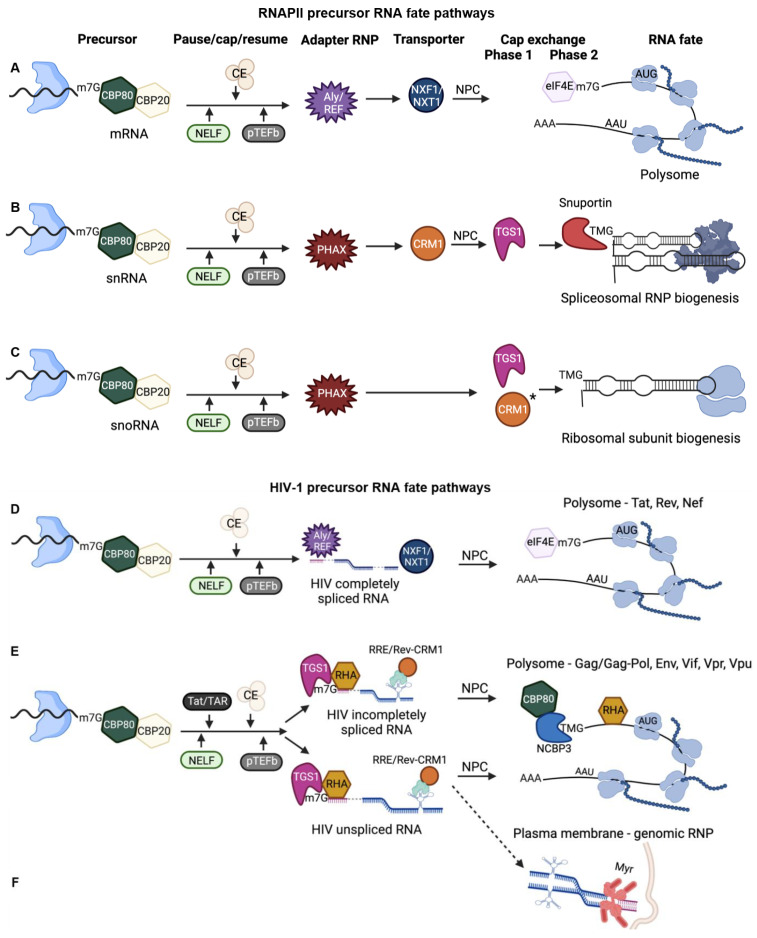
Precursor RNA fate is determined through the co-transcriptional recruitment of RNA binding proteins and subsequent rearrangement of RNP (ribonucleoprotein) components. Nascent RNA shown with paused RNA polymerase II, Blue. CE, capping enzyme. * CRM1 chaperone activity. Blue shape with black wavy line, RNA polymerase II paused on chromatin. NELF, negative elongation factor; CE, capping enzyme; pTEFb, positive transcription elongation factor b; NPC, nuclear pore complex. (**A**) Precursors of mRNA templates are completely spliced and licensed for nuclear export and CBP80/CBP20 nuclear cap-binding complex (CBC) exchange to eIF4E for canonical cap-dependent steady state translation. (**B**,**C**) snRNA and snoRNA gene products engage distinct RNA fate pathways that culminate in nucleolar trafficking and assembly into spliceosomes and ribosome. (**D**) Early HIV precursors become completely spliced mRNA templates by removal of alternative introns (dashed lines) that are licensed for the mRNA transport receptor NXF1/NXT1 and CBC exchange to eIF4E. These mRNAs form polysomes that translated Tat, Rev, Nef. (**E**) Late HIV precursors experience binding of Tat to TAR that trans-activates co-transcriptional capping and pTEFb activity. Rev binding to RRE activity results in 5′-cap hypermethylation, CRM1 nuclear export and specialized translation unaffected by mTOR. These mRNAs form polysomes that translated viral accessory and structural proteins. (**F**) The dashed line indicates a minority of unspliced RNA is bound by myristoylated (Myr, mauve bars) Gag polyprotein (mauve circles) and experienced dimerization at the plasma membrane. The dimeric RNA serves as genomic RNP that packaged into progeny virions.

Just after RNAPII initiates host gene transcription, the C-terminal domain (CTD) is phosphorylated at serine^5^ (Ser^5^) and the DRB sensitivity-inducing factor (DSIF) recruits the negative elongation factor complex (NELF) onto the chromatin [21,22,23]. The four-component NELF complex induces promoter-proximal pausing ~20–50 bases downstream of the TSS. The RNAPII Ser^5^-phosphorylated CTD and DSIF Spt5 subunit recruit the mammalian capping enzyme (CE) to the transcription start site (TSS) [24,25]. While still tethered to the CTD, the CE catalyzes the covalent attachment of a non-templated guanosine to the TSS of RNAPII nascent RNAs [26], as summarized in Figure 2.

**Figure 3:**
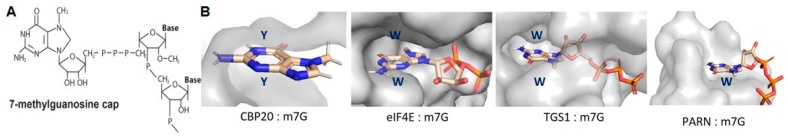
Structural basis for 7-methylguanosine (m^7^G) base recognition by cap-binding proteins. (**A**) (Transcription start site of nascent RNAPII transcripts experience 5′-5′ linkage to guanosine that is methylated at the N^7^ position to form the m^7^G-cap. (**B**) Space-filling models present the conserved m^7^G-cap binding pockets of: CBP20 of the CBP20/CBP80 heterodimer; cytoplasmic cap binding protein, eIF4E; trimethylguanosine synthase, TGS1; poly(A)-specific ribonuclease, PARN.

The guanosine cap is appended by a uniquely stable 5′-5′ linkage (Figure 3A). The CE catalyzes methylation at the N^7^ position of guanosine to form the m^7^G-cap, which is immediately bound by heterodimeric CBP20-CBP80, forming the nuclear cap-binding complex (CBC) [27,28]. Capping of the TSS is a three-step process by CE: (1) Removal of the 5′-gamma phosphate and (2) Addition of non-templated guanosine in a distinct 5′-5′ pyrophosphate linkage [29]; (3) Methylation of the non-templated guanylate by the RNMT methyltransferase domain using the methyl donor *S*-adenosyl methionine [30,31,32,33].

Within CBC, the CBP20 RNA recognition motif (RRM) sandwiches the m^7^G-cap between two aromatic residues (Figure 3B). The aromates engage the m^7^G in *pii-pii* cation-*pi* stacking interactions that stabilize the 5′-end of the nascent RNA prior to the release of the paused polymerase. That CBC-m^7^G-cap stimulates the subsequent formation of pre-initiation complexes at the promoter suggested synergy between the post-transcriptional RNPs and transcriptional initiation events back at the promoter [34].

Recruitment of the positive transcription elongation factor (p-TEFb) and its phosphorylation of Ser^2^ of CTD [35], DSIF [36,37] and NELF [38] activates the completion of the transcription cycle [24,39,40]. Cycles of CTD phosphorylation and dephosphorylation accompany the extension of the RNA precursors and modulate transcription speed [40]. Exchanges of proteins from RNAPII to the CBC-bound mRNA are reactions measured on the order of a few seconds that modulate the intramolecular refolding of RNA structures, termination of transcription and 3′-end processing [41,42,43,44]. Whereas, CBC of mRNA precursors transition to CBC-REF/ALY, sn/snoRNA precursors transition to CBC-ARS and CBC-PHAX (Figure 2) [30,31,45,46,47,48,49,50]. Even before the completion of the transcription cycle, rearrangements of RNP components accompany RNA-fate decisions.

### 2.4. CBC-m7G Precursor mRNAs Assemble Export-Competent RNPs

The CBC influences the pattern of alternative splicing by stabilizing snRNP-splice site interactions with substrate RNA [39]. CBC stabilizes the recognition of the 5′-proximal splice site by U1 snRNP, followed by the U4/U6-U5 tri-snRNP [51,52,53,54]. Thereafter, CBC-associates with REF/ALY, which transfers onto exon-exon junctions and an 3′-end processing completes the assembly of export-competent RNPs [51,52,53] (Figure 2A).

REF/ALY deposition on exon-exon junctions facilitates joining with the NXF1/NXT1 (TAP/p15) nuclear export receptor and nuclear export of CBC-bound mRNA through the nuclear pore complex (NPC) (Figure 2). In the cytosol, the m^7^G-cap experiences the exchange of CBC to eIF4E, which has a similar binding motif and engages m^7^G in an aromatic sandwich domain (Figure 3B). The exchange of CBC for eIF4E is a principal requirement for global cap-dependent translation (more below).

### 2.5. Co-Transcriptional Capping of HIV RNA Is Stimulated by Tat/TAR

HIV Tat/TAR-dependent phosphorylation of RNAP II CTD is crucial not only in promoting transcription elongation, but also in stimulating nascent viral RNA capping [55]. Phosphorylation of CTD by Tat-induced p-TEFb enhances the CE-guanylyltransferase activity and transcription elongation [55]. There is physical interaction between the CE and the C-terminal domain of Tat bound to TAR [56,57]. Whether or not the co-transcriptional stimulation of guanylyltransferase and triphosphatase activities activated by Tat/TAR influences the fate of viral transcripts remains to be fully investigated.

### 2.6. Studies Have Ascertained the Importance of HIV Cap-Proximal 2′-O Ribose Methylation to Evade Host Detection

The TSS (+1) and second transcribed nt (+2) of many mRNA precursors and some snRNA precursors experience 2′-O-ribose methylation [58,59,60,61]. Catalyzed by cap methyltransferases (CMTR), CMTR1 is recruited early in transcription to Ser^5^-phosphorylated Pol II CTD, as reviewed in [34]. These RNA modifications to host mRNAs deflect sensing by RIG-I and other antiviral RNA sensors and are considered identifiers of transcripts as ‘self’, instead of inducers of innate immune suppression [28].

The cap-specific adenosine methyltransferase phosphorylated CTD-interacting factor 1 (PCIF1) catalyzes methylation of the cap-proximal adenosine to m^7^Gpppm^6^A_m_ on host mRNAs involved in transcriptional regulation and stress response [62,63]. Moreover, PCIF1-m^6^A_m_ methylation has been shown to attenuate interferon-β–mediated suppression of viral infection. Mutation or knockout of PCIF1 suppresses vesicular stomatitis virus (VSV) and rabies virus (RABV) infection [64]. VSV and RABV mRNAs are substrates of PCIF1, indicating PCIF1 activity contributes to viral evasion of innate immune suppression. PCIF1 methylation activity on host substrates was found to restrict HIV replication [63]. Analysis of the HIV-1 TSS initially identified guanosine-guanosine as the primary sequence at the 5′ end of HIV-1 in lymphocytes [65,66,67]. Recently, an adenosine at the HIV TSS was detected infrequently [68]. Thus, PCIF1 improbably contributes to HIV evasion of innate immune suppression directly [69]. HIV accessory viral protein R (Vpr) has been shown to counter PCIF1 repression of proviruses by triggering its ubiquitination and degradation through a proteasome pathway [63]. Genome-wide screens of PCIF1-knockout cells identified several transcription factors, e.g., ETS1 and HUSH family components. PCIF1 activity on ETS1 mRNA was shown to stabilize the transcript, upregulating the ETS1 protein that repressed HIV promotor activity [69]. Vpr also counteracted transcriptional repression of proviruses by the HUSH complex that may also be regulated by PCIF1 [69]. Experiments are warranted to fully investigate PCIF1 activity on HUSH components and other HIV host dependency and restriction factors [63].

## 3. CBC Exchange Is a Precursor to Global Cap-Dependent Translation

### 3.1. eIF4E Binding to m^7^G-Cap Is Necessary for Global mRNA Translation

**Figure 4:**
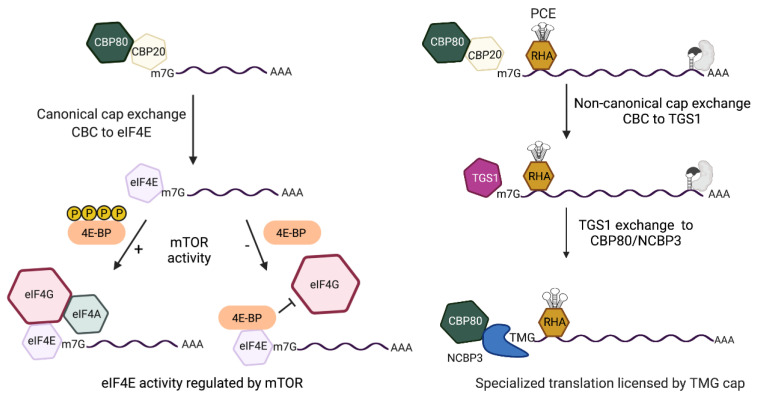
Comparison of the cap-dependent translation pathways of HIV early and late mRNAs that are licensed by m^7^G-cap or m^2,2,7^-trimethylguanosine cap(TMG), respectively. (**Left**) m^7^G-capped early mRNAs assemble nuclear cap-binding complex (CBC) composed of CBP80/CBP20. After nuclear export, CBC exchanges to eIF4E. eIF4E recruits eIF4G/eIF4A for eIF4E-dependent translation initiation that is modulated by mTOR (mechanistic target of rapamycin). Activated mTOR (+) hyper-phosphorylates 4E-binding protein (BP), blocking its interaction with eIF4E. mTOR inhibition (−) upregulates hypo-phosphorylated (hypo) 4E-BP for allosteric binding with eIF4E that blocks eIF4G interaction and promptly halts eIF4E-dependent translation. (**Right**) m^7^G-capped late mRNAs contain the Rev-responsive element (RRE) (gray RNA element) and require Rev binding (gray shape). The RRE-dependent mRNAs tether DHX9/RNA helicase A (RHA) through the post-transcriptional control element (PCE) and experience non-canonical CBC exchange to trimethylguanosine synthase 1 (TGS1). TGS1 hypermethylates m^7^G-cap to TMG-cap and exchanges to CBP80-NCBP3 for pecialized translation that is unaffected by mTOR.

Cell proliferation and cell cycle progression are modulated by fluctuations in the eIF4E-dependent translational pathway [70,71]. Global control of eIF4E-dependent translation is finely tuned through the evolutionarily conserved serine/threonine kinase, mechanistic target of rapamycin (mTOR) [72]. Amongst the substrates of mTOR are the eIF4E binding proteins (4EBPs). Inhibition of mTOR upregulates hypo-phosphorylated 4EBPs (hypo-4EBPs). These proteins serve as allosteric inhibitors of eIF4E-bound to m^7^G-cap mRNA and block interaction with eIF4G, forestalling eIF4E interactions to form the trimeric eIF4F mRNP (Figure 4).

The eIF4F complex is composed of eIF4E cap-binding protein, eIF4G scaffold protein and eIF4A helicase, as reviewed in [73]. Hypo-4EBPs promptly downregulate eIF4F translation initiation on a global scale [71] (Figure 4). Hypo-4EBP are activated by nutrient deprivation, viral infections, reactive oxygen species (ROS) and other stressors [74]. Allosteric inhibition of eIF4E by hypo-4EBPs is a trigger for cell cycle progression from G1/S phase to the G2/M phase [75]. The recovery of hyper-phosphorylated 4EBPs (hyper-4EBPs) by activated mTOR stimulates the progression of the cell cycle from mitosis to G1 phase by liberating eIF4E to nucleate the trimeric eIF4F complex [76]. eIF4G interaction with eIF3 triggers binding of the small ribosomal subunit with the eIF2.GTP.tRNA_i_^MET^ ternary complex, eIF1/1A and eIF5 for ribosome scanning and AUG recognition, 60S joining and polypeptide elongation.

The global footprint of eIF4F activating cell growth underlies its frequent hyper-regulation in malignancy and its antagonism by successful pathogens [76]. Functional genomics analyses have documented reprogramming of the eIF4F-dependent translatome is a hallmark of neoplasms [76]. Causes have been attributed to mutational inactivation of 4EBP, constitutive signaling by mTOR and dysfunction of eIF4E and eIF4G homologs [77]. Stimuli that dysregulate 4EBP perturb cellular homeostasis on a global scale. Such perturbation of eIF4E-dependent translation attributable to Vpr in HIV—associated malignancy will be important to investigate in future studies.

### 3.2. eIF4E-like Proteins Substituting for eIF4E Activate Translation Unaffected by mTOR

An adaptive translation response to stressors was recently attributed to an independent protein family of m^7^G-cap binding protein, eIF4E3 [78]. Vertebrate eIF4E3 is not an isoform of eIF4E and instead represents an independent family of cap-binding protein [79]. Vertebrate eIF4E3 shares 25% sequence identity with vertebrate eIF4E family members (eIF4E-1, -2) and exhibits differences in the cap-binding pocket that convey activity trans-dominant to eIF4E [80]. Like CBC, eIF4E sandwiches m^7^G-capbetween two aromatic residues. Elegant mutagenesis experiments characterized the atypical mode eIF4E3 sandwiching the 5′-cap with one tryptophan (Trp) and a cysteine (Cys) residue [80]. The eIF4E and eIF4E3 binding affinity for methylated guanosine-cap is 1000-fold greater than to guanosine [80,81].

During physiological downregulation of eIF4E, eIF4E3 engages the eIF4G scaffold protein for recruitment of eIF3. Two studies of eIF4E3 mutation or elimination have identified tumor suppressor activity attributable to the Trp/Cys cap-binding contacts [80,82]. Pertinent to virologists, physiological or drug-induced mTOR inhibition downregulates eIF4E, but does not necessarily eliminate cap-dependent translation because of initiation RNPs composed of eIF4E-like cap-binding proteins, e.g., eIF4E3, and the alternative, cap-independent initiation (more below).

### 3.3. Alternative RNA-Protein Interactions Support Cap-Dependent Translation

Growing literature documents additional protein families [83,84,85,86,87,88,89,90,91] can tether the 43S ribosomes for scanning to AUG start codons (reviewed in Borden et al.) [92]. The middle domain of eIF4G conveys the interaction with eIF3-bound to the 43S ribosomal subunit-eIF2.GTP.tRNA_i_^MET^ ternary complex, eIF1/1A, eIF5. Some adaptor proteins have a middle domain like eIF4G (MIF4G-like domain) [93,94] that tether eIF3 complexes to mRNA templates [95]. DAP/p97 is a MIF4G-like protein that has been shown to tether eIF3-complexes for cap-dependent translation without eIF4E. In quiescent cells, the poly(A)-specific ribonuclease (PARN) provides the m^7^G-cap binding protein and DAP/p97 tethers the eIF3-ribosome without eIF4G [87,90]. Another RNP for cap-dependent translation during physiological mTOR inhibition and eIF4E depletion is DAP5-eIF3d, which may function interchangeably with eIF4E-eIF4GI on selected mRNAs [90]. Moreover, direct binding of eIF3d to m^7^G-cap has been shown to tether ribosomal subunits for scanning of c-jun mRNA [91].

The MIF4G domain-containing protein, CTIF, has been shown to be unique in directing translation of CBC-capped mRNAs that have yet to exchange CBC for eIF4E, including the replication-dependent histone mRNAs [96,97]. Recently, CTIF has been shown to tether CBC-bound mRNP to the perinuclear region of the nuclear pore through the nucleoporin-binding protein DDX19B [98]. The perinuclear retention of CTIF by DDX19B repressed translation of CTIF-CBC mRNPs. Notably, CTIF-CBC mRNPs released to the cytoplasm were shown to form eIF4E-independent polysomes, demonstrating the translatability of CBC-bounded mRNAs. The CTIF-DDX19B interaction was shown to play an inhibitory role in nonsense mediated decay [98,99,100]. CTIF is an inhibitor of HIV Gag synthesis [99], which may also be attributable to perinuclear trapping [98].

### 3.4. Alternative RNA-Protein Interactions Support Cap-Independent Internal Ribosome Entry

Ribosome recruitment to mRNAs under conditions eIF4E is downregulated also may be attributable to internal ribosome entry site (IRES) [101,102]. Indeed, in viruses that lack m^7^G-cap, e.g., picornaviruses, internal ribosome entry and translation initiation without ribosome scanning were shown to be absolutely required for viral protein synthesis [103,104]. The IRES of naturally uncapped picornavirus RNAs were the first shown to tether the ribosome in proximity to the start codon for translation initiation without ribosome scanning (encephalomyocarditis virus, poliovirus) [103,104]. The significance of IRES activity in hosts, and therefore translation initiation without ribosome scanning, was shown to belong to post-transcriptional gene networks that carry out cell death and survival decisions [103,105]. The global scope of IRES activity relative to eIF4E3 and other widespread forms of translation initiation involving ribosome scanning remains to be completely quantified in hosts. The list of exceptional RNPs for ribosome recruitment during physiological downregulation of eIF4E has been growing [92]. 

The case for translation initiation by both linear and discontinuous ribosome scanning of select HIV RNA has been documented, e.g., bicistronic HIV env [106,107]. Notably, the cap-dependent recruitment of ribosomes and scanning to the AUG start codon is a fundamentally distinct process from the cap-independent process of internal ribosome entry to engage the start codon [103,104,108,109]. There is compelling evidence supporting the presence of an IRES within HIV tat 5′-UTR [110]. HIV IRES activity provides a cap-independent explanation for viral protein translation that is responsive to arrest of the cell cycle and other conditions downregulating eIF4E [110,111,112,113,114,115,116,117,118,119]. Elegant study of unspliced HIV RNA has identified a conditional IRES activity in the 5′-UTR that is negatively regulated and becomes de-repressed by oxidative stress [120]. The extensive study of cap-independent ribosome entry to engage the start codon of HIV mRNAs and the activity of IRES trans-acting factors are topics of recent authoritative reviews, e.g., [121,122,123,124].

## 4. Unique RNP Assembly Pathways Drive the Post-Transcriptional Fates of TMG-Capped sn/snoRNAs and Anomalous HIV RNAs

### 4.1. CRM1-Dependent Nuclear Export of CBC Bound m^7^G-snRNA Leads to Cap Hypermethylation, Retrograde Transport and Nucleolar Trafficking

The scaffolding function of CBC on snRNA precursors underlies its’ dynamic progression to CBC-NELF to CBC-ARS2 and to CBC-phosphorylated nuclear export adapter (PHAX) (Figure 2B). PHAX tethers the snRNA cargo to the CRM1 nuclear export receptor. PHAX de-phosphorylation in the cytosol releases CRM1 from the snRNA cargo and CRM1 serves to recycle CBC through an interaction of Importin-β with the CBP80 subunit of CBC [125].

**Figure 5:**
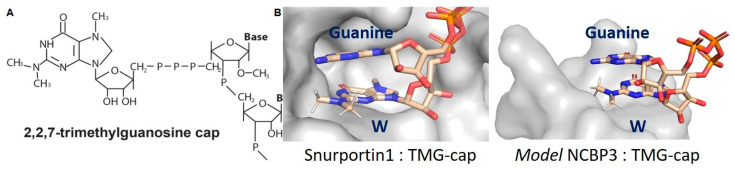
The hypermethylation of the m^7^G-cap eliminates capacity for Watson-Crick base pairing and for CBC/eIF4E/TGS1 binding. (**A**) The ^2,2,7^-trimethylguanosine (TMG-cap) appended to the 5′-terminus of RNA. (**B**) Space-filling model of the TMG-binding pocket of Snurportin1 and NCBP3 modeled on the structure of PARN. In each, the TMG-cap exhibits base stacking with the aromatic ring of tryptophan (W) in the polypeptide and guanine in the cognate RNA.

The TGS1-bound m^7^G-cap undergoes separate and distributive reactions that transfer methyl groups to the guanine N^2^ position from the methyl donor S-adenosyl-L-methionine (SAM/AdoMet) [126]. TGS1 catalysis produces two S-adenosyl-L-homocysteine (AdoHcy) molecules and the N^2,2,7^-methyl guanosine cap (TMG-cap) (Figure 5A). TGS1 affinity for the snRNA m^7^G-cap was conveyed by the aromatic amino acid sandwich motif, whereas TMG-cap is incompatible. To date, the only solved vertebrate protein structure that is compatible with the TMG-cap structure is Snurportin1 [127]. Snurportin1, also known as the snRNP-specific import adaptor, binds TMG-cap through aromatic rings of Try and base stacking by guanine of the snRNA strand (Figure 5B).

The Snurportin1-TMG-capped snRNA complex binds the Importin-β receptor for retrograde transport of the processed snRNA [128,129]. Thereafter, CRM1 promotes release and nuclear export of Snurportin1. The TMG-capped snRNP traffics through nucleoli and guides the 2′-O-methylation or pseudouridylation of snRNA and rRNA, which is critically important for the assembly of spliceosomal and ribosomal RNPs [130].

### 4.2. snoRNA m*^7^*G-Cap Experiences Hypermethylation in the Nucleus Prior to Nucleolar Trafficking

Whereas snRNA-caps undergo hypermethylation in the cytosol, snoRNA-caps experience TGS1 activity in the nucleoplasm [131,132]. Following completion of the transcription cycle and CBC binding by PHAX, snoRNA traffic to nuclear Cajal bodies and CBC exchanges to TGS1 for cap-hypermethylation [133] (Figure 2C). Again, CRM1 is involved, but instead of nuclear transport activity, CRM1 provides chaperone activity for the exposure of Nop58 nucleolar localization sequence (NoLS) [134]. The covalent modification of CRM1 by leptomycin B was shown to trap TGS1 with snoRNPs and preclude nucleolar transport [11,135]. The nucleolar TMG-capped U3 snoRNA function in ribosome assembly and involve U4/U6-U5 snoRNP and U1 snRNP [136,137]. In sum, TGS1 activity on snRNA and snoRNA requires disparate interaction with CBC-PHAX and CRM1. The unique pathways for retrograde transport of snRNAs and the nuclear retention of snoRNAsculminate in the mutual assembly into nucleolar RNPs that have cooperative activity on the fate of mRNAs.

### 4.3. HIV Infectivity Is Diminished by Downregulation of HIV TMG-Cap

TGS1 and Rev were identified as binding partners in yeast two hybrid screens, and co-precipitation studies demonstrated HIV Rev/RRE-dependent mRNAs are substrates of TGS1 in the nucleus [138]. Exogenous expression of TGS1 was shown to increase expression of the Rev/RRE-dependent viral US RNA significantly and this activity was abrogated upon replacement of Rev/RRE by the constitutive transport element (CTE). Reinforcing the importance of TGS1-Rev interaction, TMG-cap is enriched in HIV US and SS mRNAs, but not MS HIV RNAs (Figure 2D–F). The downregulation of TGS1 has been shown to abolish TMG-cap from US/SS viral RNAs, eliminate specialized translation of viral proteins, and diminish the infectivity of normalized virions by several orders of magnitude [20]. TGS1 and the host factors supporting the activity of the HIV TMG-cap are crucial host dependency factors that warrant further investigation [20].

### 4.4. Selected Host and Viral mRNAs Are Substrates for Specialized Translation and TGS1

JunD is among the host mRNAs that exhibit specialized translation [139]. JunD is a component of the heterodimeric AP1 transcription factor, e.g., JunD/Fos, whose DNA binding activity is triggered by oxidative stress and enables cells to cope with ROS and restore homeostasis [140]. In response to oxidative stress, selenoproteins involved in antioxidant defense exhibit select upregulation of translation (Gpx1, Gpx4, TR1, SelS, SelK and Sps2) [141]. Selenoproteins serve as oxidative stress reducers, anti-inflammatory actors and facilitators of wound healing [142]. Selenoprotein translation is needed in cells to cope with oxidative stress and re-balance ROS/scavenger equilibrium [142].

Wurth et al. captured TMG-capped selenoprotein mRNAs using TMG-specific antiserum in sensitive RNA immunoprecipitation and microarray experiments [143]. RT-qPCR and TGS1 siRNA downregulation validated TMG-capped selenoprotein mRNA were substrates of TGS1. Moreover, the selenoprotein, Gpx1, was shown to require TMG to support efficient translation. Experiments are warranted to validate Gpx1 is a substrate for the specialized translation pathway used for JunD and HIV protein synthesis. A very recent study has shown the selenoproteome to be altered slightly by HIV infection [144] and the significance to HIV/AIDS warrants investigation.

Selenium is an essential micronutrient incorporated into selenocysteine for selenoprotein translation [145]. Clinically, selenium supplementation is protective to people living with HIV (PLWH) [146]. Both HIV and selenium deficiency cause CD+ T cell chronic activation and proliferation that culminates in T cell exhaustion and apoptosis and the combination may exacerbate T cell decline [147]. Meta-analysis of HIV infected patients posited selenium was required for selenoprotein translation and delayed the onset of immune cell depletion [148]. Selenium deficiency in untreated HIV-infected patients was associated with a lower CD4 T-cell count [149].

## 5. TGS1 Is Tethered to HIV-1 Rev/RRE-Dependent RNAs by Host Nuclear RNA Helicase

### 5.1. Nuclear RNA Helicase Supports Recruitment of TGS1 to HIV RNA and Cap Hypermethylation Licenses Specialized Translation Unaffected by mTOR

Investigation has begun to fully explain why the abrogation of TMG-cap significantly diminishes HIV-1 proliferation [20]. The HIV TMG-cap was shown to assemble CBP80/NCBP3 polysomes that initiate structural/accessory protein synthesis unaffected by mTOR [20]. To engage TGS1, HIV 5′-UTR requires the binding of nuclear DHX9/RNA helicase ADHX9 (RHA) (Figure 2E). RHA is the host dependency factor of HIV that also stimulates translation of other retroviral and select host mRNAs through its recognition of the structure of the post-transcriptional element (PCE) in these 5′-UTRs [150,151,152,153,154,155,156]. HIV 5′-UTR was identified to contain PCE that is recognized by RHA to facilitate polyribosome association and stimulate cap-dependent translation [157,158]. RHA-RNA co-precipitation and PCE reporter assays identified HIV TAR-PolyA regions and the three-way junction structure of the PBS-segment within the 5′-UTR were necessary for PCE activity [158,159].

Homology modeling of RHA based on crystal structure of *Drosophila* Maleness (MLE) protein suggested that the RNA binding interface in the conserved double-stranded RNA binding domain (dsRBD) II is likely partially sequestered by the helicase core domain and thus, the dsRBD I was implicated with the major role in target RNA recognition [160]. Nuclear magnetic resonance and isothermal calorimetry data showed RHA’s dsRBD I interacts with double-stranded residues at the base of the PBS-segment [160,161]. Structural analysis of RHA dsRBD I association with PBS-segment identified shape-specific recognition at the three-way junction of the primer activation stem, tRNA-like element stem and tRNA annealing stem [160]. The shape-specific recognition by RHA is abrogated by a single A140C structural mutation that results in an elongated PBS-segment [160,161].

RHA-PCE interaction has been identified to tether TGS1 to the HIV RNA for hyper-methylation of the m^7^G cap [20]. The downregulation of RHA or A140C structural mutation of PCE eliminates the hypermethylated-cap from the Rev/RRE-dependent transcripts [20]. Without TMG-cap, eIF4E-dependent translation mRNPs assembled and were downregulated by mTOR inhibition. The TMG-cap licenses viral mRNAs for specialized translation unaffected by mTOR.

### 5.2. Bimodal Translation Control Is Significant to Persistent Infection

HIV structural protein translation continues during G2/M arrest and other conditions eIF4E-mediated translation is inhibited [75,117]. By emulating the TMG-cap ascribed to the noncoding sn/snoRNA, TMG cap licenses the Rev/RRE-dependent mRNA for cap-dependent ribosome recruitment and scanning supported by an unprecedented collection of nuclear proteins in the CBP80/NCBP3-RHA RNP [20,139]. Evidence for the function of NCBP3 in complex with CBP80 has been provided [139] and homology modeling of NCBP3 was built due to its sequence similarity with PARN [162]. PARN is a 3′-UTR binding protein with binding specificity for polyadenylate residues and transient interaction with m^7^G-cap that results in exonucleolytic decay. The cap binding pocket in NCBP3 is more open than that of PARN, leaving enough space for the two methyl groups at N^2^ of the guanosine in the TMG-cap (Figure 5B). Similar to Snurportin1, NCBP3 has an aromatic amino acid (Trp) to support the stacking of TMG-cap, as well as an open space to accommodate additional stacking of the adjacent purine of TMG-cap in the RNA (Figure 5B). The interaction of NCBP3 with TMG-cap is speculated to be stabilized by guanine at the HIV TSS, thus mimicking the guanne stabilization of snRNA TMG-cap with Snurportin1. The details of these molecular interactions require further structural studies.

**Figure 6:**
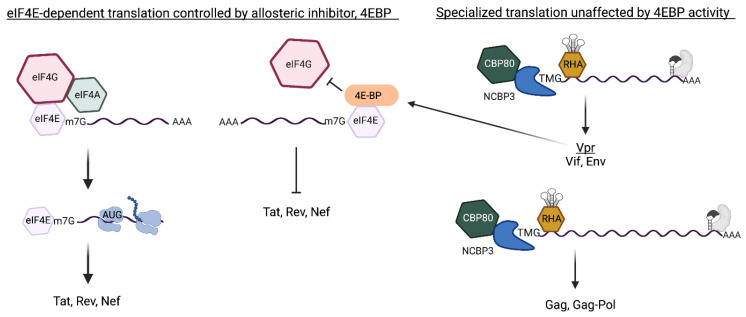
The HIV early and late mRNAs engage independent translation pathways that interdependently control the expression of viral regulatory proteins and structural/accessory proteins. (**Left**) HIV early mRNAs are fully processed mRNAs that undergo canonical eIF4E-dependent translation to Tat, Rev and Nef. Hypo-phosphorylated (hypo) 4E-BP allosterically binds eIF4E and blocks eIF4G interaction and halts the translation initiation. (**Right**) Trimethylguanosine (TMG)-cap of the Rev/Rev-responsive element (RRE)-dependent mRNAs licenses specialized translation independently of eIF4E activity. HIV unspliced and singly spliced (US and SS) mRNAs engage Rev at the RRE (gray protein binding gray RNA element) and subvert canonical CBC exchange to eIF4E and engages CBP80/NCBP3 Vpr upregulates hypo-4E-BP [75] and inhibits eIF4E-dependent translation of host proteins and Tat, Rev, Nef. The specialized translation of the Rev/RRE-dependent TMG-capped mRNAs endures until lack of Tat/Rev activity curtails biosynthesis of the US/SS RNA [20]. The downregulation of the TMG-capped mRNAs encoding viral accessory/structural protein and attenuates virus proliferation [20].

As discussed, the translation of MS mRNA is dependent on eIF4E and halted by mTOR inhibition (Figure 6, left panel), whereas the specialized translation pathway of the Rev/RRE-dependent mRNAs is unaffected by mTOR (Figure 6, right panel). Productive HIV replication in lymphocytes has been shown to downregulate eIF4E in a manner similar to mTOR inhibition [20,75]. The HIV accessory protein Vpr was shown sufficient to upregulate hypo-4EBP (Figure 6, arrow) [75],which halts the de novo synthesis of Tat and Rev [20]. Pre-existing HIV TMG-capped mRNAs in CBP80/NCBP3 mRNPs maintain specialized translation [20].

Molecular clones that abolish TMG-cap experience mono-modal translation dependent on eIF4E and replicate poorly [20], suggesting the bimodal translation control conferred by CBP80/NCBP3 was beneficial for maintaininginfected cells [20]. The CBP80/NCBP3 RNPs manifest a host mechanism that bypasses mTOR inhibition for translation of host stress response proteins [136]. The co-opt of specialized translation by HIV maintains translation of virion proteins despite mTOR inhibition [20,117]. Viral activation of hypo-4EBP through Vpr, uncouples the precursor–product relationship between Tat and Rev and the US/SS mRNAs and serves to attenuate virus proliferation [20].

### 5.3. Unexpected Findings from Study of HIV Unspliced RNA by In Situ Hybridization-Proximity Ligation Assay Protocol (ISH-PLA)

Studies have documented HIV Rev/RRE-dependent translation mRNPs exclude eIF4E [20,75,100]. Consensus for the preferential association of the polysomal HIV US mRNA with CBP80 developed through immunoprecipitation studies and visualization by ISH-PLA [18,73,84]. Consistent with preferential association of CBP80 with the unspliced RNA, the overexpression of CBP80 increased Renilla synthesis from chimeric gag-renilla US RNA [100]. The change was due to an increase in both cytoplasmic accumulation and translation of the US mRNA, suggesting a dearth of endogenous CBP80 had been rescued.

Results with the ISH-PLA protocol resonated with prior observations that Rev/RRE interaction bolstered nuclear export and the translation of HIV unspliced RNA [163,164]. ISH-PLA detected HIV US RNA in the cytosol of Rev-deficient cells [100] attributed to a minority RNP that was unique from the CBP80 translation RNP [165]. It will be interesting to ascertain whether TMG-capped RNA is a component of the minority RNP in Rev-deficient cells.

Identifying RNP components that license a minority HIV RNP, could inform the long-standing issue authentic HIV RNA in productively infected lymphocytes exists in mutually exclusive RNPs directed to translation and packaging [166]. Studies of productively infected lymphocytes have documented HIV genomic RNA packaged into virions does not require prior experience as template for Gag translation, and thus may represent a distinct RNP [167]. Rev-deficient, but CTE-containing US RNAs lack TMG-cap [138] and therefore lack specialized translation, yet exhibit efficient encapsidation [168]. Experiments are warranted to validate HIV TMG-capped US RNA is poorly packaged into virions.

In summary, it is now well-established that HIV MS RNPs carry out the maturation steps engaged by host protein-coding mRNPs (Figure 2A,D). However, HIV precursor RNAs undergoing the co-transcriptional trans-activation by Tat/TAR interaction and Rev/RRE-dependent maturation take on properties of host non-coding RNAs (Figure 2E). They experience hypermethylation of m^7^G-cap (Figure 2B,C), assemble RNPs that transit nucleoli [169,170] and experience CRM1-dependent nuclear export. In departure from the noncoding RNAs, the TMG-capped Rev/RRE-dependent mRNAs also experience specialized translation through an initiation pathway in common with host JunD, an AP1 transcription factor [139]. JunD activity during oxidative stress helps cells to rescue redox homeostasis [140].

Redox homeostasis has been a pillar in the construction of HIV Cure strategies to manipulate latently infected cells in aviremic patients [171,172,173]. Oxidative stress has been proposed to send proviruses into deep latency [173]. Until the identification of the CBP80/NCPB3-specialized translation pathway that is licensed by TMG-cap on selected mRNAs [20,139], host translation during oxidative stress was attributed largely to cap-independent initiation [143]. The recent data suggest hosts utilize specialized translation to deal with oxidative stress. It is possible that HIV co-opt of host specialized translation enables viral persistence concomitant with an effective host response to stress.

## 6. Issues, Experimental Questions, Closing

### 6.1. TMG-Cap Expands the Conformation Space of the HIV-1 5′-UTR

While investigating HIV 5′-UTR determinants for competitive packaging, Ding et al. reported that the base pairing between m^7^G-cap and cytosine (C) 57 is an important determinant (Figure 1B) [19]. They also reported TSS heterogeneity disrupts the m^7^G-C57 pairing and postulated the unpaired m^7^G-cap was available to engage eIF4E for translation [19]. Isolation of HIV US RNA from virions or polysomal mRNPs was performed and TSS heterogeneity was identified. The 5′-end of virion RNA was inferred m^7^G-cap-G, while polysomal mRNA was inferred m^7^G-cap-GGG.

**Figure 7:**
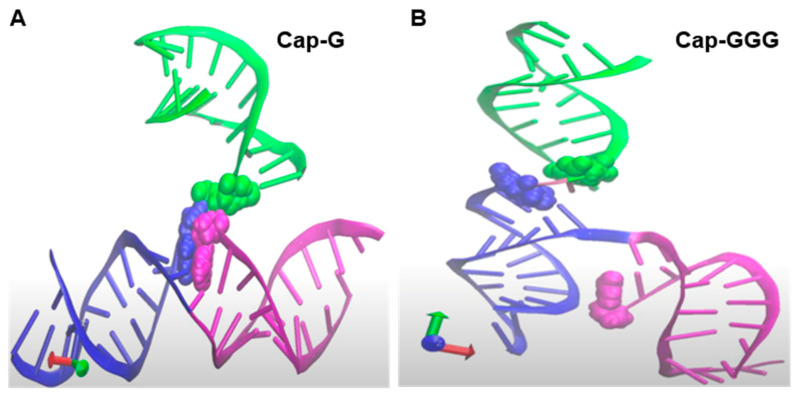
Predicted tertiary structure context of the HIV Capof the HIV ^NL4−3^ 5′-UTR given transcription start site heterogeneity of one guanosine (G) or GGG. Zoom-in on the junction of TAR (magenta)-PolyA (blue)-U5 (green) helices. Cap, magenta atom; G104, blue atom and U105, green atom. (**A**) Cap-G at the 5′ terminus. (**B**) Cap-GGG at the 5′ terminus repositioned Cap ~26 Angstroms from the junction of PolyA-U5. Rotation of these structures is provided in Video S1.

Using experimentally determined 5′-UTR input constraints and 30 million iterative Monte Carlo Simulations, tertiary (3D) models have been predicted on the simRNA platform [174]. Molecular dynamic visualization of the conformational differences between Cap-G and Cap-GGG were reviewed. Consistent with Ding et al. [19] Cap-G was sequestered at the confluence of TAR, PolyA and U5 helices (Figure 7A). In 3D, TAR-PolyA-U5 junction was compact and 5′-G and C57 were the Watson–Crick pair at the end of TAR. In addition, C58-G104 basepaired, constricting the 5′-G at the confluence of TAR-PolyA-U5 (Figure 7A and Video S1). With the addition of guanine residues at the TSS (Cap-GGG), significant structural change was apparent at the central hub of TAR-PolyA-U5 (Figure 7B and Video S1). Notably, the Cap was separated from the junction of PolyA-U5. The net result was the junction looks strong, rigid and well defined andCap was unpaired for cofactor interaction. Consistent with this model, an HIV TMG-cap is incompatible with base pairing since hypermethylation of m^7^G-cap disrupts the Watson–Crick interface, however TMG is unsuitable for eIF4E interaction [80,81]. As discussed above, cell-based experiments have documented HIV TMG-cap engages NCBP3/CBP80 heterodimer for translation in a Rev/RRE-dependent manner [20]. Taken together, in-cellulo data, in-solution analyses and in silico modeling converge on the hypothesis that TSS heterogeneity modulates HIV translation control, as well as the short- and long-range interactions in the HIV 5′ UTR that regulate genome dimerization and packaging [68,175,176]. Future studies are warranted to document the TSS of the TMG-capped RNA. Ultimately, structural study of the CBP80/NCBP3 TMG-cap interface may provide new clues for therapeutic intervention of HIV proliferation.

### 6.2. Closing

While constitutive specialized translation of HIV accessory proteins and virion proteins has been shown, the supply of US/SS mRNA templates is dictated by mTOR regulated translation of Tat and Rev [20]. Virological experiments have provided strong evidence virion proliferation is controlled by the bi-phasic cap-dependent translation (attributable to m^7^G and TMG-caps). As summarized in Figure 6,HIV Vpr-induced mTOR inhibition serves to attenuate the expression of viral regulatory proteins, which then downregulates the US/SS mRNAs encoding structural/accessory proteins. Experiments are warranted to measure the conservation of HIV specialized translation licensed by the TMG-cap in virus from patients and the closely related HIV-2.

Other questions include: Does TMG-cap contribute to masking HIV infection from innate sensing? Where is the recruitment of the NCBP3/CBP80 cap-binding complex taking place? Does the stimulation of co-transcriptional capping by Tat-bound to TAR alter the m^7^G-CBC interaction to foster TGS1 activity, and what is the role of Rev or RRE in relation to TGS1 methyltransferase activity? Does TMG-cap interfere with U1 snRNP recruitment or otherwise subvert the splicing cycle, or contribute to any stage of CRM1 nuclear export or genomic RNA packaging. Does TMG-cap experience nucleolar trafficking in common with TMG-snoRNAs? What coordination, if any, is there in the nucleolar trafficking of TGS1 and Rev/RRE mRNPs, especially in light of the observation that HIV-1 Rev variants defective in nucleolar trafficking produce poorly infectious virions [170]. Molecular plasticity in the HIV RNA World is breaching the perceived distinctiveness between the RNP components that drive the fate of non-coding-RNAs and protein-coding RNAs.

## Supplementary Materials

The following supporting information can be downloaded at: https://www.mdpi.com/article/10.3390/v14050935/s1, Video S1. Anomalous HIV-1 RNA, how cap-methylation segregates viral transcripts by form and function. Rotations of the three-dimensional visualizations show differences in the orientation of the HIV-1 5′-Cap with the junction of the helical TAR, PolyA, U5 segments. Zoom-in taken from the model of the Dimer-prone 5′-UTR (356 nucleotides) [174] on the Cap site (magenta) near the junction of TAR (magenta); PolyA (blue); U5 (green). G104, blue atom and U105, green atom (Left) Cap-G. Cap appended to the transcription start site (Cap-G) was positioned close to the central hub of TAR-PolyA-U5 helices and engaged in Watson-Crick base pairing (G-C), as summarized in Figure 1; (Right) Cap-GGG. Cap is unpaired and separated from the junction of PolyA-U5 by ~26 Angstroms. For perspective, the widest measurement of the structure is 202 Angstrom.
